# Comprehensive Communication for a Syndemic Approach to HIV Care: A Framework for Enhancing Health Communication Messages for People Living with HIV

**DOI:** 10.3390/ijerph22081231

**Published:** 2025-08-07

**Authors:** Sarah E. Sheff, Vanessa Boudewyns, Jocelyn Coleman Taylor, Hannah Getachew-Smith, Nivedita L. Bhushan, Jennifer D. Uhrig

**Affiliations:** 1 RTI International, Research Triangle Park, NC 27709, USAnbhushan@rti.org (N.L.B.); uhrig@rti.org (J.D.U.); 2 Centers for Disease Control and Prevention, Atlanta, GA 30329, USA

**Keywords:** syndemic, HIV care, health communication, framework

## Abstract

Despite the increasing adoption of a syndemic approach in HIV research, few health communication campaigns have used a syndemic approach in messaging to improve health outcomes for persons living with HIV (PWH). This paper introduces a framework for practitioners and researchers developing health communication messages in support of a syndemic approach to HIV care for PWH in the United States. Grounded in insights from a review of counseling and psychosocial interventions that demonstrated significant positive effects on HIV clinical outcomes, the C4H Framework emphasizes four components: compassion, comprehensive messaging, capacity-building, and coordination. Compassion ensures that messages resonate with individuals experiencing the intertwined challenges of HIV, substance abuse, and mental health issues. Comprehensive messaging integrates a holistic view of the barriers faced by PWH. Capacity-building empowers individuals to effectively engage with and act upon health information. Coordination promotes alignment between stakeholders and resources to ensure consistent and supportive messaging. The C4H Framework bridges the gap between research and practice, offering a foundation for crafting effective communication messages that resonate with individuals facing the complex challenges inherent in HIV syndemics. Future research should explicitly test the effectiveness and acceptability of messages developed using the C4H Framework with people living with HIV.

## 1. Introduction

The term syndemic refers to the synergistic interaction of two or more health conditions that amplify each other’s negative effects within adverse social, environmental, or economic contexts, thereby contributing to increased disease burden and health inequities [1]. Medical anthropologist Merrill Singer coined the term to describe the interplay between the HIV epidemic; social crises (e.g., poverty, chronic stress, racism, homophobia); and other health conditions like diabetes, hypertension, tuberculosis, substance abuse, and other sexually transmitted infections (STIs) [1]. Central to identifying an HIV syndemic is the biological synergism that results in poorer HIV clinical outcomes. Without this synergy, a health issue would be viewed as merely a risk factor for HIV. Singer’s foundational work focused on the substance abuse, violence, and AIDS (SAVA) syndemic, highlighting that successful interventions must address the mutually reinforcing components of the syndemic rather than tackling the three epidemics independently [2].

Researchers have moved beyond the biomedical concept of comorbidity, which historically focused on distinct disease categories, to a more comprehensive understanding of how disease burden and health disparities disproportionately impact specific communities [3]. Syndemic approaches to HIV prevention and care prioritize the whole person by addressing the combined effect of health conditions and social determinants of health, such as racism, homophobia, and poverty. These comprehensive approaches are deemed essential for ending the HIV epidemic in the United States [4].

Despite the increasing adoption of the syndemic perspective, ambiguity about what constitutes a truly syndemic-focused intervention remains [5,6,7]. Effective interventions often include multilevel and integrated strategies that not only address multiple health conditions concurrently, but also address interconnected social, economic, and environmental factors that exacerbate these conditions [8,9,10,11,12]. For example, Gilbert et al. [12] reviewed interventions that co-targeted SAVA outcomes and noted that they tended to be multilevel, integrated interventions, including individual, group, and community-level components to address the biological, behavioral, and structural mechanisms driving the SAVA syndemic among women who use drugs. Conversely, some interventions minimally integrate a syndemic perspective by simply screening for or managing co-occurring psychosocial health problems that are risk factors for syndemics [12].

Initially, our study aimed to review the existing literature on health communication campaigns (i.e., mass media or social marketing campaigns) designed to simultaneously address interrelated syndemic factors and improve HIV clinical outcomes (CD4 count, HIV viral load, engagement/retention in HIV care, antiretroviral [ARV] initiation, and adherence) among people living with HIV (PWH) in the United States over the past decade. However, despite broad search criteria and an extensive literature review (728 initial results), we identified no studies focused on communication campaigns using a syndemic approach targeting HIV clinical outcomes for PWH (see Appendix A for details about the initial literature review). Instead, we identified 16 intervention studies and 14 formative or qualitative research studies that employed health education, counseling, or psychosocial methods delivered directly by trained professionals (or peers) to individuals or small groups (hereafter referred to as counseling and/or psychosocial interventions). These interventions primarily addressed HIV care alongside syndemic cofactors such as (1) substance abuse (drugs and alcohol, including tobacco); (2) mental health issues; (3) substance abuse and mental health issues; or (4) COVID-19 (Despite a comprehensive search strategy intended to capture a variety of health conditions, we did not identify any interventions or formative communication work related to Mpox, tuberculosis, hepatitis C, other bacterial or fungal infections, other STIs, or intimate partner violence [including physical, sexual, and psychological violence] that focused on improving HIV syndemic cofactors and clinical care outcomes for PWH).

## 2. Developing Health Communication Messages to Support a Syndemic Approach to HIV Care

**Figure 1:**
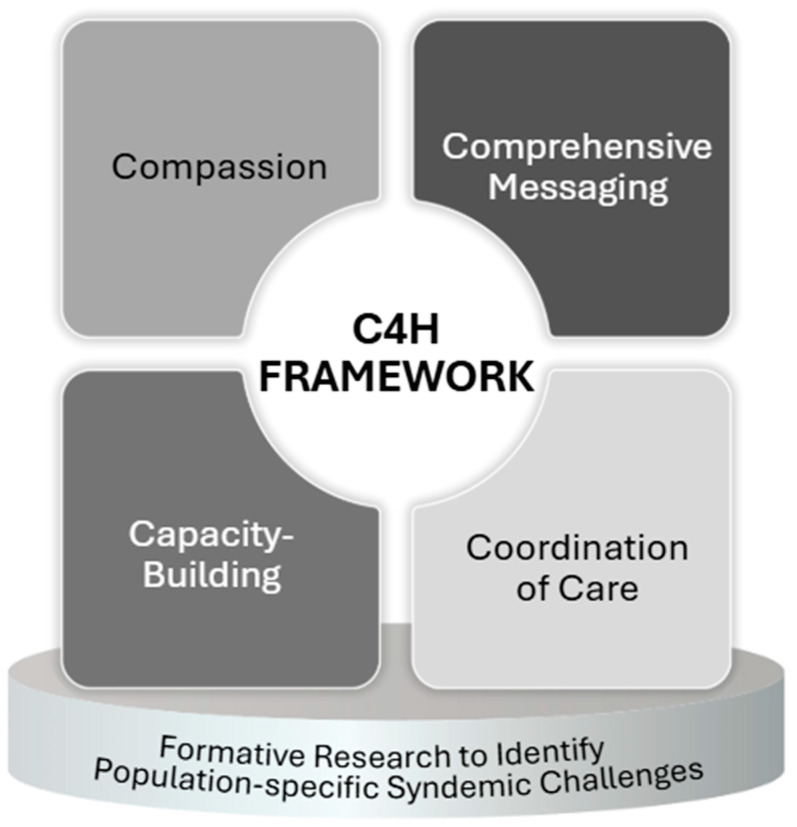
C4H Framework for Developing Health Communication Messages to Support a Syndemic Approach to HIV Care.

Given the lack of published evaluations of communication campaigns using messages that support a syndemic approach to HIV care, we extracted insights from successful counseling and/or psychosocial interventions that align with the foundational elements of communication campaigns to bridge the gap and provide a framework for message development. We focused specifically on insights related to the content and components of interventions applicable to message development (i.e., context, message delivery, and engagement duration). By synthesizing these insights, we identified key components critical to improving HIV clinical outcomes. We systematically analyzed these interventions using thematic synthesis to identify recurring effective strategies. Specifically, to identify the core components of the C4H Framework, a senior team member systematically reviewed the abstraction matrix for studies demonstrating statistically significant effects on HIV clinical outcomes. The reviewer closely examined two key abstraction fields: (1) authors’ stated recommendations for future interventions and research gaps, and (2) our team’s extrapolations about implications for future message development, testing, and formative research. Through thematic analysis of these fields, recurring themes emerged consistently across successful studies, highlighting compassion (empathetic, stigma-reducing approaches), comprehensive messaging (holistic, integrated strategies), capacity-building (skills development and self-efficacy), and coordination (integration and linkage to care) as essential components. These emergent themes formed the basis of the preliminary C4H Framework (see Figure 1).

The following describes each of the four components in the framework and suggests considerations for crafting health communication messages for PWH in support of a syndemic approach to HIV care, as well as highlighting some providing hypothetical example messages to illustrate how principles of the C4H Framework could be reflected in future health communication messages targeting PWH experiencing syndemic conditions. Although these examples were drafted for illustrative purposes, most are adapted from or informed by messages our team has previously developed and vetted in collaboration with CDC, many of which were subsequently tested with members of the intended audience as part of formative research. These descriptions and accompanying examples are intended as illustrative guidance for future message development.

Compassion. Health communication messages targeting syndemic vulnerabilities—such as HIV and substance abuse—should be intentional about the tone they use. Messages should be supportive, understanding, and nonjudgmental to create a safer space for individuals to engage with the content. PWH often face multiple vulnerabilities due to syndemic factors such as substance abuse, mental health issues, and social determinants of health like poverty and discrimination. Shame, in particular, can lead to disengagement and withdrawal from care-seeking behaviors [13]. Therefore, adopting a nonjudgmental tone in communication messages is critical.

Unlike individualized counseling sessions, mass media messages lack the personalization of face-to-face interventions. Therefore, conveying compassion in a concise and accessible manner that resonates with a broad audience is important. Messages that convey empathy and compassion can play a significant role in reducing stigma and shame, helping to build trust and encourage individuals to seek necessary health services. For instance, a proof-of-concept pilot intervention for PWH and active substance use disorders demonstrated the feasibility and acceptability of using individual therapy sessions paired with daily compassionate self-statements (e.g., “I am more than just a junky”) sent via text message. These messages specifically addressed internalized stigma and shame, which positively impacted HIV outcomes, including viral load and ARV adherence [14]. Health communication messages can replicate this approach by reinforcing positive self-worth and acknowledging the lived experiences of PWH.

However, due to the brevity and wide broadcast nature of mass media, communication messages should be carefully framed to ensure compassionate, relatable content without unintentionally marginalizing audiences. The effectiveness of this compassionate approach in more controlled settings, like those seen in the Attonito et al. study [15], suggest that well-designed health communication messages, grounded in empathy, can similarly influence positive behavioral outcomes.

Comprehensive Messaging. Although health communication campaigns inherently lack the individualized attention of counseling interventions, they can adopt a holistic and multifaceted approach by addressing the broader social, environmental, and economic factors that exacerbate syndemic conditions. Effective messages must consider the whole person, recognizing the additive effects of health conditions (e.g., HIV, substance abuse, mental health issues) within the context of adverse social environments such as poverty, discrimination, and lack of access to health care. Messages should clearly communicate these interconnected challenges, encouraging a more-coordinated and system-wide understanding of the factors at play.

Research demonstrates that connecting HIV care with related health services (such as mental health support or substance abuse treatment) in communication messages may help audiences recognize the comprehensive support available. For instance, dental care motivated PWH with substance use disorders to engage with their HIV provider, demonstrating how interconnected needs (medical documentation to access dental services necessary for restoring tooth loss) created a point of entry to HIV care [16]. Although communication messages cannot replicate the individual-level engagement that one-on-one interventions like Powers et al. [16] offer, these messages can promote the availability of integrated services across various health domains (e.g., HIV care, substance abuse treatment, mental health support).

Additionally, bundling related topics into health messages—for instance, connecting HIV care with other health services like mental health counseling or substance use treatment—may help audiences recognize the full scope of services available to support their well-being. The strategy of bundling [17] related services may provide an effective model for syndemic-focused communication. However, research highlights the importance of thoroughly testing bundled messages for effectiveness and audience receptivity [18].

In addition, messages should acknowledge the daily challenges faced by PWH, including instability in housing, telephone service, and internet access. Employing strategies such as role modeling and testimonials from trusted peers and promoting positive peer relationships can enhance the effectiveness of the message by making it relatable and trustworthy. For example, the Chicago Department of Public Health’s 2021 rebranding to the Syndemic Infectious Disease Bureau reflects an integrated approach to address HIV, hepatitis B, hepatitis C, and tuberculosis together, signaling the need for comprehensive communication strategies [19]. Similarly, the U.S. Department of Health and Human Services’ HIV.gov blog post on the HIV and Mpox syndemic underscores the importance of coordinated, holistic communication strategies addressing intersections between syndemic cofactors like mental health and homelessness, Mpox, HIV, and other STIs [20].

Capacity-Building. Capacity-building interventions are critical to improving the self-efficacy and decision-making of PWH as they navigate the challenges posed by syndemics. Two studies addressing the HIV and substance abuse syndemic reported statistically significant improvements in ARV initiation and adherence [21] and HIV viral load outcomes [22]. These interventions highlight the importance of skill-building strategies such as goal-setting, self-efficacy enhancement, and positive peer relationships. Similarly, several studies addressing the HIV and mental health syndemic, which also yielded positive HIV outcomes, underscored the importance of building skills such as mood monitoring, behavioral activation, reduction in negative thinking, and medication management [23,24,25,26].

Health communication messages should leverage these insights by promoting self-efficacy, adaptive coping, and resilience through messages highlighting achievable goals and acknowledging small victories, such as adherence to antiretroviral therapy (ART) or regular attendance at primary care visits. Highlighting incremental successes empowers individuals to take ownership of their health and motivates them to continue positive behaviors. For instance, messages like “You’re doing great—every dose of ART brings you closer to better health!” reinforce the idea that every action counts and that progress is attainable.

Incorporating digital health interventions into HIV syndemic communication can further enhance capacity-building by providing personalized support, guiding individuals through small, actionable steps toward improved health outcomes. This is consistent with guidance from CDC and other authorities recommending that campaigns segment audiences and adapt messaging for priority populations while maintaining overall campaign coherence. Mobile or electronic health tools and apps offer opportunities for real-time engagement, tailored medication reminders, mood tracking, decision-making exercises, and problem-solving skills. Meiksin et al. [27] systematically reviewed e-health interventions targeting sexual health, substance use, and mental health among men who have sex with men, identifying three key approaches: cognitive- or skills-based techniques, self-monitoring, and cognitive therapy. These digital approaches often improve health outcomes by boosting self-efficacy, improving one’s capacity to overcome challenges and enhancing self-regulation through self-monitoring features that trigger reflection and foster behavioral adjustment. Although digital tools may not fully address structural syndemic factors like discrimination, they can help address psychosocial and structural-level challenges related to mistrust or stigma and help reduce barriers to care through anonymity and accessibility [28,29].

Finally, health communication messages should also consider how to build resilience, defined as the ability to respond adaptively to adversity [30]. Research has shown that resilience plays a moderating role in reducing the negative impact of syndemics on health outcomes. For example, resilience has effectively mitigated the combined effects of substance abuse, violence, and HIV on mental health outcomes [30]. During the COVID-19 pandemic, resilience helped individuals cope with heightened stress, anxiety, and substance use while maintaining HIV care engagement [31]. Digital interventions fostering resilience among young Black gay and bisexual men demonstrate the feasibility of embedding resilience strategies into syndemic-focused communication campaigns to enhance individuals’ engagement with health services and HIV care [32]. Messages emphasizing incremental progress, adaptive coping, and overcoming of challenges are critical components for supporting resilience in PWH.

Coordination of Care. Addressing HIV syndemics requires more than one-time exposure to health communication messages. Long-term change hinges on sustained, coordinated efforts that connect individuals with ongoing support, resources, and services. Given the multifaceted nature of syndemics, coordinated care helps streamline services, simplifying access for individuals with complex needs. For example, Connecticut’s *Ending the Syndemic Initiative* advocates integrated responses to HIV, STIs, substance use disorder, and viral hepatitis through stakeholder collaboration [33].

The literature shows that sustainable improvements in syndemic outcomes require partnerships with local resources, health departments, and organizations capable of delivering integrated and long-term care [12,16,34,35]. For example, McKinnon et al. [36] note that in-person case management and outreach interventions are most consistently associated with improved outcomes, such as increased ARV medication prescribing, health care utilization, and positive clinical outcomes. A notable limitation of health communication campaigns is their inability to offer personalized follow-up or continuous interaction. Although health communication messages effectively raise awareness of the benefits of connected or coordinated care, influence attitudes and beliefs, and provide clear calls to action (such as seeking information or resources about coordinated care), they cannot directly guide people through each step of the health care system or fully address the myriad of social and structural barriers.

Therefore, health communication messages should explicitly connect individuals with local services providing continuous support. For example, a services locator widget [37] available on the U.S. Centers for Disease Control and Prevention’s Let’s Stop HIV Together website allows users to enter their ZIP code to acquire information (website, address, phone number and directions) about organizations in their area that provide prevention, testing and treatment for HIV, STIs and Hepatitis C; the Mpox vaccine; family planning; substance use treatment; mental health services; housing assistance; and more [38]. Facilitating these connections helps to ensure that individuals who respond to health communication messages are connected to resources that provide access to sustained care.

Because of the interconnected nature of HIV, other health conditions, and social and structural challenges inherent in HIV syndemics, messages designed to support a syndemic approach to HIV care should align with multiple framework components. Table 1 provides examples of health communication messages drafted to align with the C4H Framework to support a syndemic approach to HIV. These messages are not intended for immediate implementation or tailored to any specific population or literacy level; rather, they serve as examples demonstrating potential alignment with the four framework components. Future work would require formative research, including audience testing and health literacy assessment, to tailor messages appropriately for intended populations and delivery contexts (e.g., mass media campaigns, clinic posters, digital health interventions).

## 3. Contextualizing the C4H Framework: Formative Research to Identify Population-Specific Challenges Within a Syndemic

Before applying the CH4 Framework, researchers must conduct formative research to understand how the HIV syndemic of interest is influenced by social determinants of health, identify the resulting challenges that communication messages must address, and determine how to most effectively reach and engage the intended audience. For example, PWH often encounter compounded stigma, unstable housing, economic hardship, limited access to care, and difficulties accessing care, all of which are exacerbated by co-occurring conditions such as substance abuse, mental health issues, health complications from low ART adherence, and other chronic health conditions [39,40,41,42]. Additional challenges for messaging might include low technological literacy, heightened privacy concerns, medical mistrust, limited familiarity with mental health therapies, and comfort-seeking mentality [14,43,44]. Messages cannot effectively embody compassion, comprehensiveness, capacity-building, or coordination if they do not accurately reflect the context of the syndemic and the lived realities of the intended audience.

Admittedly, communication messages alone cannot resolve all challenges faced by individuals experiencing an HIV syndemic, particularly structural-level barriers. Although communication messages can increase awareness of available services, their impact may be restricted by fragmented, under-resourced health care systems and polices that limit integrated care [45]. Nonetheless, researchers must clearly identify the population-specific barriers faced by PWH experiencing the syndemic before developing messages. Doing so ensures that any proposed strategies are relevant, targeted, and impactful, ultimately contributing to improved clinical outcomes and quality of life for PWH.

### Directions for Future Research

Recent literature reviews on HIV syndemics have focused exclusively on interventions grounded in counseling [46] and psychosocial approaches [47,48], underscoring the prevalence of community-based interventions over communication efforts in addressing the complex challenges of HIV syndemics. The notable absence of HIV syndemic communication studies in the literature indicates a pressing need for future research to both inform the development of and evaluate messages designed to support a syndemic approach to HIV care. Ideally, teams crafting messages should leverage multidisciplinary collaboration, including expertise in substance use and mental health. This approach exemplifies how team science can enhance the integration of diverse perspectives and expertise, thereby improving the effectiveness of messages [49]. Whether communication messages can replicate some of the supportive, educational, and motivational aspects of effective counseling and/or psychosocial interventions for HIV syndemics remains an open question. Future research should explicitly test messages that are developed using the C4H Framework to assess perceived effectiveness before broader dissemination and campaign evaluation efforts in the United States.

The literature on HIV syndemics demonstrates that, although local contexts shape syndemics [50], many contributing factors—such as poverty, marginalization, and stigmatization—are common across diverse communities, rural or urban alike. Health communication messages can raise awareness about these shared challenges and steer individuals toward local resources and services for targeted, sustained support. Ideally, health communication messages should be integrated components of broader, multilevel public health interventions designed to comprehensively address syndemics. This comprehensive approach includes collaborative, on-the-ground efforts to simultaneously address interconnected health conditions and adverse social and structural factors [51].

## 4. Conclusions

There is a critical gap in the literature regarding the effectiveness of communicating about HIV care using a syndemic approach. Although our initial goal was to review and apply effective strategies from existing communication campaigns that used a syndemic approach, the absence of such studies in the literature necessitated shifting our strategy. Thus, we extracted and synthesized insights from successful counseling and/or psychosocial interventions that align with the foundational elements of communication campaigns to create the C4H Framework. The C4H Framework offers a structured approach that can be expanded and tailored to the diverse needs of populations in the United States affected by HIV syndemics. The framework is neither exhaustive nor definitive but can serve as a foundational starting point for the development of health communication messages that aim to improve health outcomes for PWH. We will continue to adapt and refine the framework based on empirical testing with the goal of ensuring its effectiveness across diverse contexts and populations.

## Figures and Tables

**Table 1:** Health Communication Messages Crafted Using the C4H Framework to Support a Syndemic Approach to HIV Care.

Example Message	Framework Alignment
“You have the power to navigate challenges and create positive change. Commit to your HIV care journey by addressing barriers head-on and finding strategies to overcome them. Your dedication to staying in care, managing any substance use, and nurturing your mental health can improve your overall health. A case manager can connect you with other support, such as housing, transportation, health services, health insurance coverage, and substance use disorder treatment. In most cases, HIV treatment is free! Find services near you. [Link to services.]”	Capacity-building Compassion Comprehensive messaging Coordination of care
“Life can be challenging, and daily chaos can often distract us from focusing on our health and well-being. You have the power to navigate these challenges. Make a plan to prioritize your HIV treatment, work on managing any substance use, and nurture your well-being to improve your overall health. Find a provider in your community who can support you on your journey. [Link to providers.]”	Capacity-building Compassion Coordination of care
“Choose progress over perfection. Build positive relationships and surround yourself with a supportive community. Take charge of your well-being, align your choices with your goals, manage any substance use, and prioritize your HIV treatment. Small changes can have a big impact on your health and well-being. Find services in your community. [Link to services.]”	Capacity-building Comprehensive messaging Coordination of Care
“You possess inner strength and resilience. Celebrate progress along your journey. By taking care of your mental health, you make it easier to manage your HIV.”	Capacity-building Compassion Comprehensive messaging
“Life’s challenges can sometimes get in the way of HIV care. But you are stronger than your challenges. You may face roadblocks, but you can always find an alternate route. Help is there. You can do this!”	Capacity-building Compassion
“Prioritize both your mental well-being and your HIV care. You have the power to keep track of your medications, practice self-care, and foster open communication with your care team.”	Capacity-building Comprehensive messaging
“We know it’s not easy, but you deserve support for both HIV and substance use. Treatment can make a difference, and you don’t have to face it alone.”	Compassion Comprehensive messaging
“Taking care of your mind is just as important as taking your HIV meds. Both help you stay strong and healthy. Explore free mental health and HIV care resources in your area today! [Link to services.]”	Comprehensive messaging Coordination of care

## Appendix A. Literature Review Methods

We searched PsycINFO, PubMed, and Web of Science databases to find peer-reviewed studies addressing our research questions. To identify relevant studies, we developed a comprehensive search strategy using keywords related to our key intersecting health problems, also known as syndemic cofactors: *communicable diseases*, *Monkeypox (Mpox)*, *COVID-19*, *bacterial and fungal infections*, *STIs*, *mental health issues*, *substance abuse (drugs, alcohol, and tobacco)*, and *intimate partner violence (including physical, sexual, and psychological violence)*. We included terms such as *intervention*, *campaign*, *messag**, *advertisement**, *formative research*, *formative interviews*, *formative work*, and *social marketing* to capture studies focused on interventions and campaigns aimed at addressing HIV syndemics. We also included terms like *HIV-positive*, *people living with HIV*, and *people with HIV* to ensure the inclusion of studies specifically addressing individuals living with HIV. Our search strategy also included terms related to HIV treatment and care.

To further enrich our database search, we hand-searched and reviewed seminal works, including those published before 14 December 2012, that cited Merrill Singer’s *Introduction to Syndemics* handbook. We also examined the bibliographies of foundational publications and systematic reviews for studies not initially identified in database searches. Some studies that were not included in the PsycINFO, PubMed, and Web of Science database searches were added after an additional review of the bibliographies of foundational publications and systemic reviews. Formative studies that preceded their corresponding intervention studies were also added for review.

Two team members independently reviewed all 728 titles and abstracts from the search results to determine eligibility for full-text review. We restricted the studies to those published in English and conducted in U.S. populations and settings and excluded correlational studies^2^ and commentaries. This paper describes key findings from the remaining 16 intervention studies, 14 formative or qualitative research studies, and 9 systematic reviews of studies aiming to improve HIV syndemic cofactors and clinical care outcomes for PWH.
